# Underestimated prognostic value of depression in patients with obstructive coronary artery disease

**DOI:** 10.3389/fcvm.2022.961545

**Published:** 2022-12-02

**Authors:** Quanjun Liu, Han Yin, Cheng Jiang, Mingyu Xu, Yuting Liu, Anbang Liu, Haochen Wang, Bingqing Bai, Fengyao Liu, Lan Guo, Huan Ma, Qingshan Geng

**Affiliations:** ^1^Department of Cardiology, Guangdong Cardiovascular Institute, Guangdong Provincial People's Hospital, Guangdong Academy of Medical Sciences, Guangzhou, China; ^2^School of Medicine, South China University of Technology, Guangzhou, China; ^3^Department of Cardiac Rehabilitation, Guangdong Cardiovascular Institute, Guangdong Provincial People's Hospital, Guangdong Academy of Medical Sciences, Guangzhou, China

**Keywords:** depression, coronary artery disease, left ventricular dysfunctions, tissue doppler imaging, prognosis

## Abstract

**Objective:**

The aim of this study was to explore the different predictive values of depression among patients with different cardiac systolic function levels.

**Methods:**

Four hundred eighty-three consecutive patients with obstructive coronary artery disease (CAD) were included the depressive state was assessed using the Chinese version of the Patient Health Questionnaire 9 (PHQ-9). Depression was defined as have depressive symptoms with a PHQ-9 score ≥5. The level of cardiac systolic function was classified as left ventricular ejection fraction (LVEF) ≥50 and <50%.

**Results:**

Over a median of 26.2 months, 421 patients completed the follow-up and experienced 101 major adverse cardiovascular events (MACEs), 45 non-cardiac rehospitalizations, and 17 deaths. Predictors for clinical outcomes in patients with different cardiac systolic function levels were not the same. For participants with preserved LVEF, depression was associated with increased risks for cardiovascular events and composite outcomes. However, when focusing the whole population, predictive values of depression for MACEs, non-cardiac rehospitalizations, and composite endpoints all dropped. Receiver operating characteristic (ROC) analyses further confirmed that depression was the one of the main predictors for all clinical outcomes. With the combination of other simple features, area under curve (AUC) could reach 0.64–0.67.

**Conclusions:**

Inconsistent with the general impression, depression is found to have a closer linkage with clinical outcomes in CAD patients with preserved LVEF rather than in those with decreased LVEF. These findings appeal for more attention on CAD patients with depressive symptoms and comparatively normal LVEF. Including psychological factors may be a good attempt when constructing risk prediction models.

## Introduction

Depression is a highly prevalent risk that worsens the prognoses of patients with established coronary artery disease (CAD) (1). Compared to a <10% prevalence in general population, ~20–40% of CAD patients would have a comorbid condition of depression (2). According to so far the largest meta-analysis from Meijer et al. (3), depression is associated with a 2.7-fold increased risk of cardiac-related death and a 2.3-fold increased risk of all-cause death in the next 2 years after an acute myocardial infarction (MI). Similar phenomena have also been witnessed in patients with stable CAD (4, 5) and heart failure (HF) even after the adjustment of variables for disease severity (6).

However, there is disagreement among researchers about whether depression is only a reflection of worsening physical conditions and whether the elevated risk of poor clinical outcomes is caused by the inadequate adjustment to disease severity (7). One way to possibly clarify this controversial issue is to explore differences in the predictive value of depression between patients with different cardiac function levels. Although the idea has been mentioned in prior research (8), no related outcomes have been reported.

In our prior research (9) and previous literature (10), it has been revealed that having depressive symptoms and left ventricular ejection fraction (LVEF) are two of the main predictors for cardiovascular prognosis in CAD patients. However, since the worsening of cardiac function is often accompanied with depression-like symptoms of fatigue and psychomotor retardation and also the deterioration of renal function, which might have interfered the reliability of the predictive models for cardiovascular prognosis given the possible collinearity of variables.

Therefore, in a prospective cohort of obstructive CAD patients, we attempted to explore the predictive values of having depressive symptoms in patients with different cardiac systolic function levels. Through these comparisons, we aimed to figure out the real influence of depressive symptoms on prognosis to deepen the understanding of depressive symptoms in CAD.

## Materials and methods

### Participants

This study was a *post-hoc* exploratory analysis based on the follow-up outcomes of a prospective cohort of obstructive CAD patients. It originated from a cross-sectional study on the psychological statuses of inpatients with admitting diagnoses of CAD. In the cross-sectional study (11), 705 consecutive inpatients with main admitting diagnoses of CAD and no urgency for emergency revascularization therapy or intensive care in Guangdong Provincial People's Hospital were surveyed using the Patient Health Questionnaire (PHQ-9) and Generalized Anxiety Questionnaire-7 Scale (GAD-7) between October 2017 and January 2018. All patients were warranted to be surveyed in a comfortable condition under the supervision of a well-trained psycho-cardiologist on the day before undergoing coronary angiography (CAG). Based on CAG outcomes, medical records, and discharge diagnosis, the patients were further classified into the non-CAG group, the epicardial coronary artery stenosis < 50% group, and the obstructive CAD group.

**Figure 1:**
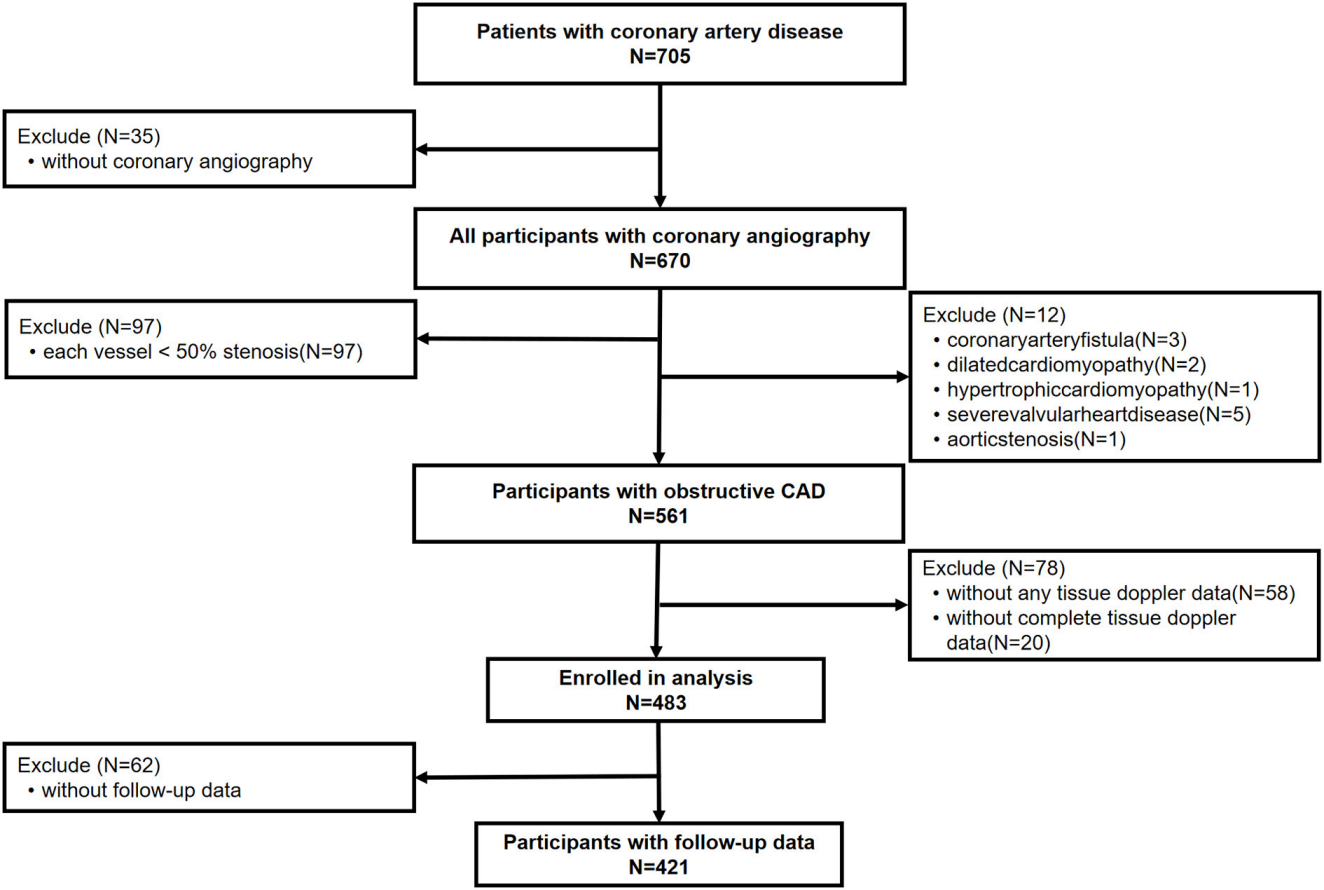
Screening flow of patients included in research. Obstructive CAD is defined as with a ≥50% stenosis in at least one of the main coronary arteries, confirmed by coronary angiography or with a history of coronary artery bypass grafting or coronary stent implantation. CAD, coronary artery disease.

This study mainly focused on the patients with obstructive CAD. Of the 573 patients included, 12 with severe valve regurgitation unlikely caused by CAD, coronary artery fistula, aortic stenosis, or non-ischemic cardiomyopathy were further excluded in consideration of the different mechanisms that caused their chest discomfort. Patients without complete conventional transthoracic echocardiography (TTE) or myocardial tissue doppler imaging (TDI) data were further excluded from the study, leaving 483 subjects in the final analyses (Figure 1).

Follow-up data were collected at the sixth-month follow-up and then yearly through a scripted telephone interview. Participants were asked about their histories of cardiac and non-cardiac rehospitalization, MI, stroke, revascularization, and death. A major adverse cardiovascular event (MACE) was defined as a composite of cardiac rehospitalization, cardiovascular death, AMI, stroke, or urgent coronary revascularization. The composite endpoint was defined as any occurrence of the events mentioned above.

The study complied with the Declaration of Helsinki and was approved by the Medical Ethics Committee of Guangdong Provincial People's Hospital with the following reference number: No. GDREC2017203H. All participants gave written informed consent before being included in the study.

### Patient health questionnaire-9

The depressive state of each patient was assessed using the Chinese version of the PHQ-9. As a valid screening tool in accordance with DSM-IV symptom criteria, it has nine items that are responded to on a 3-point Likert-type scale to capture the characteristics of their depression (12). High scores for each item represent being frequently bothered by the symptom in the last 2 weeks. According to the total score, the degree of depressive symptoms was classified as none or minimum (0–4), mild (5–9), moderate (10–13), moderately severe (14–18), and severe (12, 19–26). Depression in the analyses of this research was defined as having depressive symptoms with PHQ-9 score ≥5. The Chinese version has been demonstrated to have high validity and reliability among CAD patients (13). The PHQ-9 was found to have high internal consistency (Cronbach's α = 0.85) in our study.

### Echocardiography

Data about conventional TTE and TDI were collected from clinical records. All echocardiographic exams were performed in accordance with the recommendations of the American Society of Echocardiography using a GE Vivid E9 (GE Vingmed Ultrasound, Horten, Norway) with an M5S probe (2–4 MHz) or Philips IE33 (Philips Healthcare, Andover, Mass) with an S5-1 probe (2.5–3.5 MHz). LVEF was obtained using the modified biplane Simpson's method.

### Coronary artery stenosis severity and creatinine clearance

Coronary artery stenosis severity score was defined as the number of the three main vessels with stenosis ≥ 50%. However, a ≥ 30% luminal stenosis in the left main coronary artery would be directly classified as the highest severity level.

Creatinine clearance (CCR) was estimated using the Cockcroft-Gault formula with the value of serum creatinine tested at admission.

### Statistical analysis

Clinical characteristics were reported as frequency (percentages) for categorical variables and analyzed with chi-square or Fisher's exact tests, or for continuous variables as mean ± standard deviation if they followed a normal distribution and compared with Student's *t*-test between two groups, otherwise as median (interquartile range) and compared with Wilcoxon rank-sum test. The overall influence of depression on MACE, non-cardiac rehospitalization and composite endpoint in patients with LVEF ≥ 50% and the whole population were shown by Kaplan–Meier survival curves and compared using log-rank test. Non-normal distributed variables were divided into two categories according to the median, for example high sensitivity troponin T (Hs-TNT) ≤14 />14pg/ml, N-terminal pro-B type natriuretic peptide (Nt-proBNP) ≤180/>180 pg/ml, so as to be adjusted in analyses. Proportional-hazards (PH) assumption were examined by Log-Log survival function plots. Cox regression models including all the possibly correlated factors (*p* < 0.10) in comparisons between patients with and without certain events with forward selection methods were used to explore the main predictors for all clinical outcomes. Receiver operating characteristic (ROC) analyses were adopted to demonstrate the priority of depression for predicting all clinical outcomes. All statistical analysis was performed using SAS 9.4 software. A value of *p* < 0.05 was considered to show statistical significance.

## Results

Baseline characteristics of the enrolled inpatients are displayed in Table 1 and Supplementary Table 1. Of the 483 obstructive CAD patients, 76.6% were male with an average age of 63.62 ± 10.02 years old and LVEF of 58.2 ± 11.1%. The overall prevalence of diabetes and hypertension were 35.4 and 62.7%, respectively. The prevalence of having depressive symptoms was 38.7% (*N* = 187).

**Table 1:** Comparisons of baseline clinical characteristics according to cardiovascular prognosis in patients with different systolic function.

	**LVEF≥50%**			**LVEF<50%**			**Patients with follow-up data**		
	**Without major cardiovascular events**	**With major cardiovascular events**	***p***	**Without major cardiovascular events**	**With major cardiovascular events**	***p***	**Without major cardiovascular events**	**With major cardiovascular events**	***p***
	***N* = 270**	***N* = 70**		***N* = 50**	***N* = 31**		***N* = 320**	***N* = 101**	
Age, y	63.20 ± 9.82	63.70 ± 10.25	0.71	64.08 ± 9.96	62.65 ± 12.02	0.56	63.34 ± 9.83	63.38 ± 10.77	0.98
Male, No. (%)	202 (74.81)	49 (70.00)	0.41	42 (84.00)	22 (70.97)	0.16	244 (76.25)	71 (70.30)	0.23
CCR, ml/min	68.54 ± 19.91	64.28 ± 23.89	0.17	57.55 ± 20.57	58.77 ± 21.73	0.72	66.82 ± 20.37	62.59 ± 23.28	0.080
LDLC, mmol/L	2.92 ± 0.96	2.77 ± 0.77	0.18	2.82 ± 0.81	3.00 ± 0.91	0.36	2.90 ± 0.94	2.84 ± 0.82	0.56
HDLC, mmol/L	**0.99 ± 0.22**	**0.92 ± 0.20**	**0.018**	0.96 ± 0.25	0.91 ± 0.15	0.30	**0.98 ± 0.22**	**0.92 ± 0.18**	**0.003**
HbA1c, %	6.48 ± 1.24	6.85 ± 1.61	0.074	7.30 ± 1.90	7.49 ± 2.41	0.69	**6.61 ± 1.39**	**7.05 ± 1.90**	**0.032**
Hypertension, No. (%)	175 (64.81)	42 (60.00)	0.46	27 (54.00)	21 (67.74)	0.22	202 (63.13)	63 (62.38)	0.89
Diabetes, No. (%)	87 (32.22)	25 (35.71)	0.58	24 (48.00)	11 (35.48)	0.27	111 (34.69)	36 (35.64)	0.86
hsCRP, μg/ml	2.11 (0.78,7.35)	1.86 (0.67,6.00)	0.39	5.38 (2.04,15.30)	5.26 (2.74,28.80)	0.59	2.45 (0.82,9.10)	2.74 (0.91,7.09)	0.98
Hs-TNT, pg/ml	**12 (8, 24)**	**16 (10,51)**	**0.034**	95 (22,458)	83 (23,846)	0.54	**13 (8,37)**	**22 (12,109)**	**<0.001**
Nt-pro-BNP, pg/ml	126 (52,307)	141 (40,445)	0.57	1370 (600,3440)	1697 (655,5995)	0.45	**157 (60,483)**	**318 (56,1609)**	**0.009**
History of revascularization, No. (%)	**79 (29.26)**	**31 (44.29)**	**0.017**	19 (38.00)	9 (29.03)	0.41	98 (30.63)	40 (39.60)	0.094
Stenosis degree, No. (%)			0.22			**0.008**			**0.014**
1	62 (22.96)	10 (14.29)		**8 (16.00)**	**0 (0)**		**70 (21.88)**	**10 (9.90)**	
2	47 (17.41)	15 (21.43)		**14 (28.00)**	**6 (19.35)**		**61 (19.06)**	**21 (20.79)**	
3	161 (59.63)	45 (64.29)		**28 (56.00)**	**25 (80.65)**		**189 (59.06)**	**70 (69.31)**	
ACEI/ARB, No. (%)	195 (72.22)	52 (74.29)	0.73	41 (82.00)	26 (83.87)	0.83	236 (73.75)	78 (77.23)	0.48
mono or dual antiplatelet, No. (%)	266 (98.52)	69 (98.57)	>0.99	50 (100)	31 (100)	>0.99	316 (98.75)	100 (99.01)	>0.99
Statin, No. (%)	267 (98.89)	67 (95.71)	0.20	50 (100)	30 (96.77)	0.38	317 (99.06)	97 (96.04)	0.10
β-blockers, No. (%)	231 (85.56)	62 (88.57)	0.51	44 (88.00)	30 (96.77)	0.34	275 (85.94)	92 (91.09)	0.18
CCB, No. (%)	68 (25.19)	21 (30.00)	0.41	8 (16.00)	6 (19.35)	0.70	76 (23.75)	27 (26.73)	0.54
Anticoagulant, No. (%)	5 (1.85)	2 (2.86)	0.96	0 (0)	2 (6.45)	0.14	5 (1.56)	4 (3.96)	0.29
Furosemide, No. (%)	10 (3.70)	3 (4.29)	>0.99	19 (38.00)	13 (41.94)	0.72	29 (9.06)	16 (15.84)	0.055
LVEF, %	63.04 ± 5.08	62.67 ± 5.74	0.60	**41.52 ± 5.78**	**36.16 ± 8.48**	**0.003**	**59.71 ± 9.41**	**54.54 ± 13.98**	**<0.001**
PHQ-9 score^†^, point	**3.86 ± 3.85**	**5.84 ± 5.24**	**<0.001**	4.52 ± 4.00	3.77 ± 3.31	0.47	**3.96 ± 3.87**	**5.21 ± 4.82**	**0.011**
Prevalence of depression, %	**87 (32.22)**	**35 (50.00)**	**0.006**	26 (52.00)	12 (38.71)	0.24	**113 (35.31)**	**47 (46.53)**	**0.043**
GAD-7 score^†^, point	2.90 ± 3.13	4.39 ± 5.17	0.084	2.88 ± 3.42	2.65 ± 3.37	0.61	2.90 ± 3.18	3.85 ± 4.74	0.29
Prevalence of anxiety, %	67 (24.81)	25 (35.71)	0.067	11 (22.00)	6 (19.35)	0.78	78 (24.38)	31 (30.69)	0.21

† Scores were presented as means ± SD, however were compared using Wilcoxon rank-sum test.

LVEF, left ventricular ejection fraction; CCR, creatinine clearance; LDLC, low density lipoprotein; HDLC, high-density lipoprotein; ACEI, angiotensin converting enzyme inhibitor; HbA1c, glycosylated hemoglobin; HsCRP, high sensitivity C-reactive protein; Hs-TNT, high sensitivity troponin T; ARB, angiotensin receptor blocker; CCB, calcium channel blockers. Significant differences are shown in bold.

In the course of a median 26.2 months (740–855 days), 421 patients (87.2%) completed the follow-up and experienced 101 (24.0%) MACEs, 45 (10.7%) non-cardiac rehospitalizations, 17(4.0%) deaths, 16 (3.8%) non-fatal strokes, and 140 (33.3%) composite events. No differences in baseline characteristics were observed between all patients and those with complete datasets (Supplementary Table 1).

### Baseline characteristics and clinical outcomes in CAD patients with LVEF ≥50 and <50%

Compared to patients with obstructive CAD and preserved LVEF (≥50%) (Supplementary Table 2), patients with LVEF<50% had worse cardiac (Nt-proBNP; furosemide use) and renal function, more severe myocardial impairment (Hs-TNT), higher levels of inflammation (Hs-CRP) (all *p* < 0.001), diabetes (*p* = 0.017), and a tendency toward higher degrees of coronary artery stenosis (*p* = 0.075). However, the PHQ-9 (4.37 ± 4.30 vs. 4.57 ± 4.28, *p* = 0.50) and GAD-7 (3.26 ± 3.71 vs. 2.84 ± 3.73, *p* = 0.18) scores were not statistically different between groups. Neither were the prevalence of having depressive symptoms (*p* = 0.067) or anxious symptoms (*p* = 0.13).

Quite consistent with our common sense, clinical outcomes, especially for cardiovascular prognosis were strikingly worse in patients with LVEF<50% than those with preserved LVEF (MACE 38.3 vs. 20.6%, *p* < 0.001; death 12.3 vs. 2.1%, *p* < 0.001) (Supplementary Figure 1).

### Related factors with depression

Associations between depression and other clinical features in patients with LVEF ≥50% and the whole population were presented in Table 2. For patients with LVEF ≥50%, the significantly related factors were age (*p* = 0.017), gender (*p* < 0.001), renal function (CCR: *p* = 0.002), and GAD-7 score (*p* < 0.001); while for whole population, the most relevant factors were age (*p* = 0.017), gender (*p* < 0.001), renal function (CCR: *p* < 0.001), history of diabetes (*p* = 0.012), Nt-pro-BNP (*p* = 0.003), GAD-7 score (*p* < 0.001), and LVEF (*p* = 0.017).

**Table 2:** Comparisons of baseline characteristics between groups categorized by depression in patients with LVEF≥50% and in the whole population.

	**Patients with LVEF≥50% (*****N*** = **387)**			**All patients (*****N*** = **483)**		
	**PHQ-9<5** ***N* = 245**	**PHQ-9≥5** ***N* = 142**	***P* value**	**PHQ-9<5** ***N* = 296**	**PHQ-9≥5** ***N* = 187**	***P* value**
Age, y	62.58 ± 9.76	65.08 ± 9.99	**0.017**	62.75 ± 9.93	64.99 ± 10.04	**0.017**
Male, No. (%)	204 (83.27)	88 (61.97)	**<0.001**	246 (83.11)	124 (66.31)	**<0.001**
CCR, ml/min	69.48 ± 19.93	62.39 ± 22.89	**0.002**	68.13 ± 20.32	59.83 ± 22.37	**<0.001**
LDLC, mmol/L	2.83 ± 0.90	2.93 ± 0.94	0.30	2.83 ± 0.90	2.91 ± 0.90	0.37
HDLC, mmol/L	0.96 ± 0.20	0.99 ± 0.23	0.25	0.95 ± 0.21	0.98 ± 0.23	0.11
HbA1c, %	6.54 ± 1.32	6.59 ± 1.36	0.75	6.64 ± 1.51	6.83 ± 1.63	0.19
Hypertension, No. (%)	152 (62.04)	93 (65.49)	0.50	184 (62.16)	119 (63.63)	0.74
Diabetes, No. (%)	73 (29.80)	54 (38.03)	0.097	92 (31.08)	79 (42.25)	**0.012**
HsCRP, μg/ml	1.74 (0.78,5.87)	2.73 (0.79,7.70)	0.32	2.21 (0.83,8.08)	3.26 (0.95,8.66)	0.22
Hs-TNT, pg/ml	12 (9, 26)	13 (9, 28)	0.68	13 (9,48)	18 (10,71)	0.12
Nt-pro-BNP, pg/ml	114 (49,319)	166 (62,433)	0.081	145 (54,493)	250 (87,1080)	**0.003**
History of revascularization, No. (%)	82 (33.47)	45 (31.69)	0.72	97 (32.77)	64 (34.22)	0.74
History of CAG, No. (%)	117 (47.76)	68 (47.89)	0.96	147 (49.66)	96 (51.34)	0.59
Stenosis degree, No. (%)			0.31			0.45
1	46 (18.78)	34 (23.94)		50 (16.89)	40 (21.39)	
2	49 (20.00)	26 (18.31)		64 (21.62)	34 (18.18)	
3	150 (61.22)	82 (57.75)		182 (61.49)	113 (60.43)	
ACEI/ARB, No. (%)	172 (70.20)	108 (76.06)	0.21	216 (72.97)	143 (76.47)	0.39
Mono or dual antiplatelet, No. (%)	244 (99.59)	138 (97.18)	0.12	295 (99.66)	183 (97.86)	0.15
Statin, No. (%)	241 (98.37)	137 (96.48)	0.40	291 (98.31)	181 (96.79)	0.44
β-blockers, No. (%)	204 (83.27)	129 (90.85)	**0.038**	252 (85.14)	167 (89.30)	0.19
CCB, No. (%)	60 (24.49)	42 (29.58)	0.27	67 (22.64)	50 (26.74)	0.31
Anticoagulant, No. (%)	4 (1.63)	6 (4.12)	0.22	5 (1.69)	7 (3.74)	0.27
Furosemide, No. (%)	10 (4.08)	7 (4.93)	0.69	26 (8.78)	31 (16.58)	0.010
LVEF, %	63.05 ± 5.11	62.46 ± 5.72	0.30	59.14 ± 10.20	56.56 ± 12.23	**0.017**
PHQ-9 score^†^, point	1.84 ± 1.39	8.73 ± 4.13	**<0.001**	1.80 ± 1.41	8.53 ± 4.09	**<0.001**
Prevalence of depression, %	0 (0)	142 (100)	**<0.001**	0 (0)	187 (100)	**<0.001**
GAD-7 score^†^, point	1.91 ± 2.17	5.59 ± 4.58	**<0.001**	1.81 ± 2.07	5.34 ± 4.62	**<0.001**
Prevalence of anxiety, %	30 (12.24)	76 (53.52)	**<0.001**	31 (10.47)	94 (50.27)	**<0.001**

† Scores were presented as means ± SD, however were compared using Wilcoxon rank-sum test.

LVEF, left ventricular ejection fraction; CCR, creatinine clearance; LDLC, low density lipoprotein; HDLC, high-density lipoprotein; CAG, coronary angiogram; ACEI, angiotensin converting enzyme inhibitor; HbA1c, glycosylated hemoglobin; hsCRP, high sensitivity C-reactive protein; hs-TNT, high sensitivity troponin T; ARB, angiotensin receptor blocker; CCB, calcium channel blockers. Significant differences are shown in bold.

### Depression—predictor for MACEs

Apparently, the predictors for MACEs in univariate anaylses (shown in Table 1) in patients with different disease severity levels were not the same. The key predictors for MACEs in patients with LVEF<50% were the severity of coronary artery stenosis (*p* = 0.008) and LVEF value (*p* = 0.003). By contrast, the history of revascularization (*p* = 0.017), level of serum HDLC (high-density lipoprotein cholesterol, *p* = 0.018), and hs-TNT (*p* = 0.034), and PHQ-9 score (*p* < 0.001) constituted the main determinants for MACEs in patients with preserved LVEFs. Values of Nt-proBNP were close between those with and without MACEs in the subgroup comparisons, however had a remarkable difference in the whole population.

Multivariate analyses (Table 3) including all the possible factors in univariate analyses with a forward selection method revealed that having depressive symptoms (hazard ratio (HR): 2.01 [95% confidence interval (CI),1.27,3.19], *p* = 0.003), history of revascularization (HR: 1.62 [1.01,2.59], *p* < 0.05), and level of serum HDLC (per 0.1 mmol/L increase HR: 0.29 [0.09,0.90], *p* = 0.03) were the main predictors of MACE among patients with LVEF ≥50%; while coronary stenosis severity (HR: 1.93 [0.96, 3.84], *p* = 0.06) and LVEF value (per 10% increase HR: 0.95 [0.91, 0.99], *p* < 0.01) determined the cardiovascular prognosis in patients with LVEF < 50%. For the whole population, although having depressive symptoms, coronary stenosis severity, HDLC, and LVEF value were retained in the model, the predictive effect of depressive symptoms (HR: 1.37 [0.93, 2.01], *p* = 0.098) for MACE distinctly weakened.

**Table 3:** Predictors for MACE in patients with different systolic function using Cox regression models^†^.

**Variable**	**Crude**		**Multivariable**	
	**HR (95% CI)**	***P***	**HR (95% CI)**	***P***
**LVEF≥50%**				
Depression				
PHQ-9 <5	Ref.		Ref.	
PHQ-9≥5	1.98 (1.25,3.14)	0.004	2.01 (1.27,3.19)	0.003
**History of revascularization**				
No	Ref.		Ref.	
Yes	1.72 (1.08, 2.74)	0.022	1.62 (1.01, 2.59)	0.046
HDLC (every 1.0 mmol/L increase)	0.25 (0.08, 0.80)	0.020	0.29 (0.09, 0.90)	0.033
**LVEF <50%**				
Stenosis severity (per class increase)	2.24 (1.14, 4,41)	0.020	1.93 (0.96, 3.84)	0.057
LVEF (per 1% increase)	0.95 (0.91, 0.99)	0.009	0.95 (0.91, 0.99)	0.009
**All patients**				
Depression				
PHQ-9 <5	Ref.		Ref.	
PHQ-9≥5	1.45 (0.99, 2.12)	0.058	1.37 (0.93, S2.01)	0.098
Stenosis severity (per class increase)	1.38 (1.06, 1.81)	0.019	1.26 (0.95, 1.66)	0.071
HDLC (per 0.1 mmol/L increase)	0.34 (0.13, 0.87)	0.021	0.44 (0.17, 1.13)	0.056
LVEF (per 10% increase)	0.97 (0.95, 0.98)	<0.001	0.97 (0.95, 0.98)	<0.001

† Cox regression models with forward selection method (sle=0.10, sls=0.10) were used.

MACE, major adverse cardiovascular event; HR, hazard ratio; CI, confidential interval.

### Predictive value of depression for other clinical endpoints

**Figure 2:**
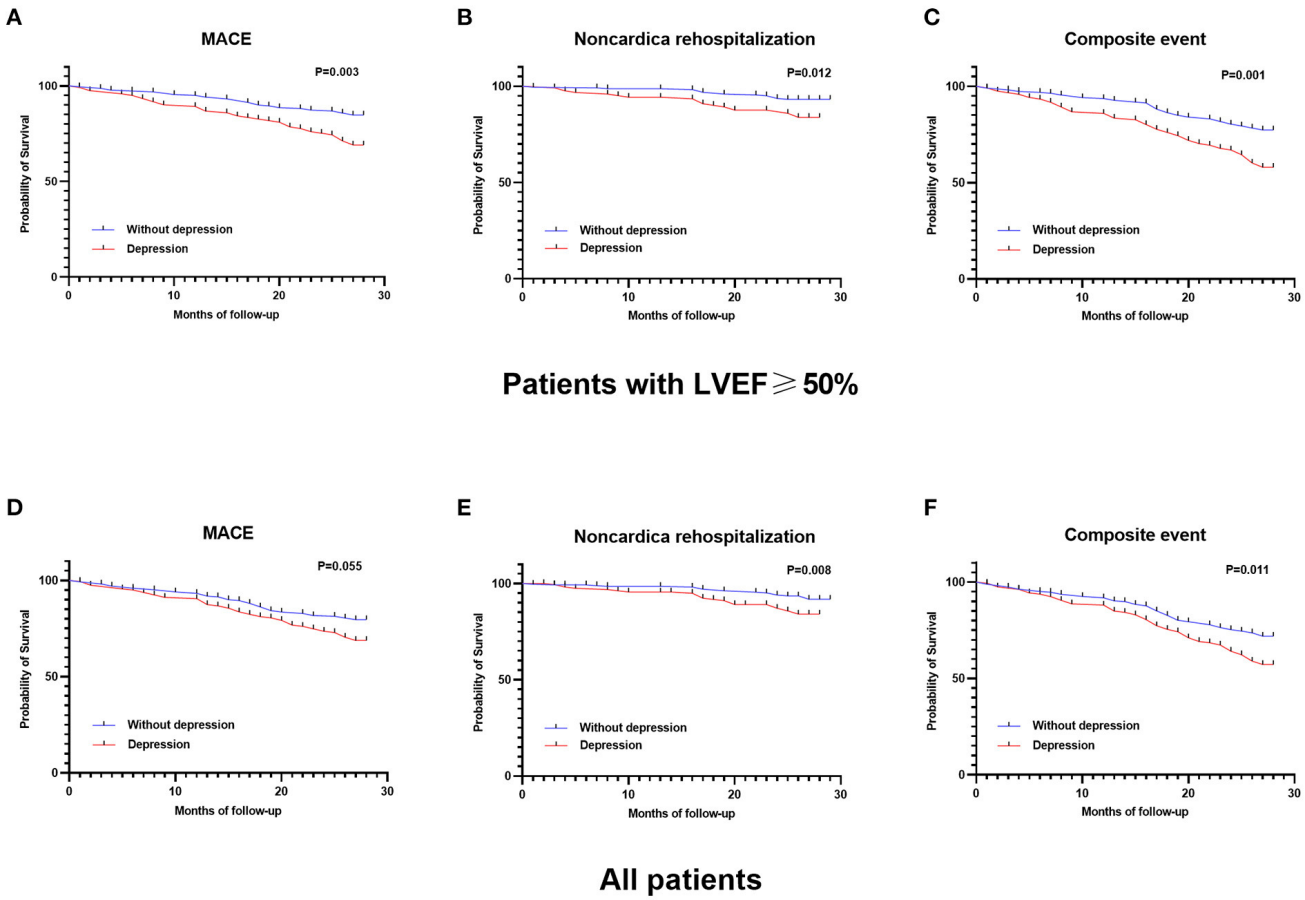
Kaplan–Meier survival analyses of depression in patients with LVEF≥50% for **(A)** MACE, **(B)** non-cardiac rehospitalization, **(C)** composite event, and in all patients for **(D)** MACE, **(E)** non-cardiac rehospitalization, and **(F)** composite event. The prognostic value of depression on all clinical outcomes were more significant in patients with LVEF≥50%.

Kaplan–Meier survival analyses (Figure 2) further demonstrated that depression was also associated with non-cardiac rehospitalization and composite events. Both the prognostic values of having depressive symptoms for non-cardiac rehospitalization (for LVEF ≥50%: crude HR 1.99 [1.05, 3.76] vs. for all: crude HR 1.65 [0.98, 2.78]) and composite events (for LVEF ≥50%: crude HR 1.75 [1.18, 2.60] vs. for all: crude HR 1.46 [1.04, 2.03]) declined when focusing on the whole population. After adjusting for confounders with Cox regression models, these trends still remianed (Supplementary Tables 3, 4).

### ROC analyses for depression in predicting clinical outcomes

ROC analyses (Table 4) demonstrated that depressive symptoms showed good predictive value for MACEs in patients with LVEF ≥50% [area under curve (AUC) = 0.59] and in the whole population (AUC = 0.56). With the addition of the other relevant clinical features, the AUC could be further increased among patients with preserved LVEF (AUC = 0.67) and in the whole population (AUC = 0.66). Similar outcomes were also observed in the analyses for non-cardiac rehospitalization and composite endpoint (shown in Supplementary Tables 5, 6). In those comparisons among the main predictors for clinical outcomes, no clinial feature demonstrated a better statistical significance than depression.

**Table 4:** Comparisons of predictive values for MACE in patients with LVEF≥50% and in the whole population.

	**ROC association statistics**			
	**AUC**	**Standard error**	**95% CI**	***P***
**LVEF≥50%**				
Depression	0.59	0.033	0.52, 0.65	ref
HDLC	0.59	0.038	0.51, 0.66	0.97
History of revascularization	0.58	0.033	0.51, 0.64	0.77
Depression + HDLC + History of revascularization	0.67	0.035	0.60, 0.74	0.007
**All patients**				
Depression	0.56	0.028	0.50, 0.61	ref
HDLC	0.58	0.031	0.52, 0.64	0.53
Stenosis severity	0.56	0.026	0.51, 0.62	0.83
LVEF	0.60	0.034	0.54, 0.67	0.27
Depression + HDLC	0.66	0.031	0.60, 0.72	0.004

MACE, major adverse cardiovascular event; LVEF, left ventricular ejection fraction; HDLC, high-density lipoprotein; ROC, Receiver Operating Characteristic Curve; AUC, area under curve; CI, confidential interval.

## Discussion

In this exploratory analysis of a prospective cohort, we demonstrated that the predictors for clinical outcomes in patients with different cardiac systolic function levels were not the same. For participants with preserved LVEF, having depressive symptoms was associated with increased risks for cardiovascular events and composite outcomes. However, when focusing the whole population, predictive values of depression symptoms for clinical outcomes all decreased. Having depressive symptoms was one of the main predictors for different clinical outcomes, after adjusting for confounders, and with combination with other clinical features, good predictive effects could be reached by predicting models.

### Depression and prognosis

Apparently, the clinical symptoms of depression characterized by fatigue and psychomotor retardation have a certain degree of overlap with HF. Considering the graded relationship between depressive symptoms and the subsequent risk of mortality and cardiovascular events (6, 14, 15) and the fact that the risk associated with depression frequently decreases after adjusting potential confounders, it is believed by some experts that depression in patients might be expressions of severe heart disease rather than a comorbid depressive disorder (1). In fact, based on our prior research (11), depressive symptoms in patients with CAD has been greatly affected by physical discomfort and this impact of the disease on mood symptoms tends to occur in patients with poor physical conditions.

However, through the exploration of the predictive values of depressive symptoms in different left ventricular systolic function levels, we confirm that having depressive symptoms is at least to a certain degree independently associated with poor cardiovascular prognosis and all-cause mortality in patients with CAD (16–18). Furthermore, the phenomenon that depressive symptoms was even more closely associated with clinical outcomes in patients with comparatively less severe cardiac function damage, implies that the importance of mood disturbance in CAD patients with relatively normal systolic function might have been underestimated in previous researches. This finding, to the best of our knowledge, has not been reported in the literature. Additionally, we noticed that depressive symptoms was also relevant to non-cardiac rehospitalization, which however was not found in patients with MI (19).

One thing should be noted is that the prevalence of having depressive symptoms in patients with MACE was lower in those with LVEF <50% (38.7%) than those with LVEF ≥50% (50.0%) (*p* = 0.24). This result may be explained by the high prevalence of depression in HF patients with preserved LVEF (HFpEF) (20). Another reason may be the misdiagnosis of mental disorders, which happens from time to time in non-obstructive CAD patients in cardiology departments (21). The symptoms like tiredness, disturbed sleep, loss of appetite caused by depression can be subjectively attributed to the heart disease by patients, which further lower the surveyed rate of depression.

In our study, cardiovascular deaths accounted for 11.4% of MACEs among the depressed patients with preserved LVEF and 41.7% of MACEs among patients with LVEF < 50%. As is known, depression is often coupled with elevated anxiety. Anxiety may help promote health-seeking behaviors (22), which, in turn, may cause the increase in rehospitalization. For patients with severe cardiac dysfunction, who usually have worse physical conditions and more severe coronary stenosis, the level of anxiety is in fact disproportionately decreased (11). This might have contributed to the lower predictive value of depression for the non-fatal outcomes. Besides, the adaptation to the frequent occurrence of discomfort and the decrease in motor ability may increase the pain threshold at which one seeks for medical help.

The more pronounced influence of depressive symptoms on clinical outcomes in patients with relatively normal systolic function also hints that antidepressant treatment could probably achieved better effect in patients with less severe disease severity. This may provide an explanation for the negative outcomes of randomized controlled trials on serotonin reuptake inhibitors (SSRIs) in heart failure (23, 24) and the positive outcomes in acute coronary syndrome from the study by Kim et al. (25) with the enrolled patients having comparatively lower disease severity levels. Non-pharmacological treatment like exercise training has been proved to improve prognosis and alleviate depression symptoms, which could be attributed be the increased initiative under better cardiopulmonary conditions in seeking medical help.

### Predicting models with depression and clinical features

Predicting the prognoses of patients with CAD has been a challenge for cardiologists. Given the fact that total cardiovascular disease burden has gradually shifted toward non-fatal outcomes in recent years, the commonly used tools such as SCORE (26), GRACE score (27), and TIMI risk score (28) may lose part of their significance.

In the current research, we explored the predictors for MACEs, non-cardiac rehospitalizations and composite endpoints in patients with different systolic function levels and found that having depressive symptoms was one of the main stable variable associated with worse prognoses (29). In accord with previous researches (30, 31), coronary stenosis severity and LVEF value were found to be another two important risk factors for predicting cardiac outcomes, and creatinine clearance (32) and sex difference (33) were associated with non-cardiac rehospitalization.

By introducing depression into the predicting models, the predictive effect could achieved a fairly good level especially in patients with preserved LVFE, through a combination of several common clinical features. This may be due to the fact that depression is not only the reflection of both somatic and psychological discomfort, but also the determinant for the health-seeking behaviors. Besides, as mentioned in our prior research, psychological factor significantly correlates with the prehospital decision delay (34).

In sum, this current research indicates that more attention should be paid to the CAD patients with prolonged depressive symptoms and preserved LVEF. Meanwhile it also enlightens us of the possibility that parameters correlated with the psychological or social states of patients may become necessary considerations when constructing a risk prediction model for non-fatal outcomes.

## Limitations

There are several limitations to this study. First of all, this study is a *post hoc* exploratory analysis of follow-up data based on a single-center cross-sectional study. However, it should be mentioned that exploring the impact of depressive symptoms on clinical outcomes is exactly the primary outcome of the prospective cohort. Besides, due to the consecutive enrolling strategy, the representative of the sample could be guaranteed. Secondly, the sample is comparatively too small, which restricted further analyses of the influence of clinical depression in patients with reduced LVEF. Depression defined in the research is different from clinical depression, which could be screened out with a good sensitivity and specificity with PHQ-9 ≥10. However, previously researches have shown that even minor depressive symptoms have significant negative effects on prognoses (35). Thirdly, many patients were excluded from the analysis due to dropout and a lack of echocardiography data, which can bring about bias. Lastly, the use of psychotropic drugs was not included in the analyses, even though only 8 in the whole population were taking antidepressant treatment.

## Conclusions

Predictive values of having depressive symptoms on clinical outcomes in CAD patients with preserved LVEF have been underestimated as compared to the results analyzed in the whole population. These findings once more stress the important influence of depression on prognosis and appeal for more attention on CAD patients with depression and relatively normal cardiac function. Including psychological or social state factors may be a good attempt when constructing risk prediction models for non-fatal outcomes.

## Data availability statement

The raw data supporting the conclusions of this article will be made available by the authors, without undue reservation. Requests to access the datasets should be directed to QG, gengqingshan@gdph.org.cn.

## Ethics statement

The studies involving human participants were reviewed and approved by the study complied with the Declaration of Helsinki and was approved by the Medical Ethics Committee of Guangdong Provincial People's Hospital with the following reference number: No. GDREC2017203H. The patients/participants provided their written informed consent to participate in this study.

## Author contributions

HY surveyed all patients in the baseline. YL, AL, HW, BB, and FL followed all patients. HY, CJ, and MX designed the study, collected, and entered data into the database. HY, QL, and CJ conducted the statistical analyses. QL, HY, CJ, and MX wrote the paper. QG, HM, and LG were the senior physicians principally responsible for the study. HM and QG revised the paper. All authors read and approved the final manuscript.

## Funding

This work was supported by grants from the Natural Science Foundation of Guangdong Province (2021A1515011118 and 2021A1515011781), start-up Funding of National Natural Science Foundation of China (Nos. 8207120182, 8207050582, and 8217142362), the Guangzhou Science and Technology Basic and Applied Basic Research Project (202102080368), and the High-level Hospital Construction Project of Guangdong Provincial People's Hospital (DFJH201811, DFJH201922, and DFJH2020003).

## Conflict of interest

The authors declare that the research was conducted in the absence of any commercial or financial relationships that could be construed as a potential conflict of interest.

## Publisher's note

All claims expressed in this article are solely those of the authors and do not necessarily represent those of their affiliated organizations, or those of the publisher, the editors and the reviewers. Any product that may be evaluated in this article, or claim that may be made by its manufacturer, is not guaranteed or endorsed by the publisher.
