# Supplementary material for: Underestimated prognostic value of depression in patients with obstructive coronary artery disease
Source: Front Cardiovasc Med. 2022 Dec 2;9:961545. doi: 10.3389/fcvm.2022.961545 (PMC9755582; doi:10.3389/fcvm.2022.961545)
Supplement: Supplementary file 1 [file Data_Sheet_1.docx]

Supplementary Material

# Supplementary Table 1

| **Table S1 Comparisons of baseline clinical characteristics between whole population and those with follow-up data.** | | |
| --- | --- | --- |
|  | All patients N=483 | Patients with follow-up data N=421 |
| Age, y | 63.62±10.02 | 63.35±10.05 |
| Male, No.(%) | 370(76.60) | 315(74.82) |
| CCR, ml/min | 64.92±21.50 | 65.80±21.16 |
| LDLC, mmol/L | 2.86±0.90 | 2.89±0.91 |
| HDLC, mmol/L | 0.96±0.22 | 0.97±0.22 |
| HbA1c, % | 6.72±1.56 | 6.71±1.54 |
| Hypertension, No.(%) | 303(62.73) | 265(62.95) |
| Diabetes, No.(%) | 171(35.40) | 147(34.92) |
| HsCRP, μg/ml | 2.51(0.89,8.39) | 2.46(0.87,8.08) |
| HsTNT, pg/ml | 15(9,58) | 14(9,62) |
| Nt-pro-BNP, pg/ml | 187(63,708) | 180(60,587) |
| History of revascularization, No.(%) | 161(33.33) | 138(32.78) |
| Stenosis degree, No.(%) |  |  |
| 1 | 90(18.63) | 80(19.00) |
| 2 | 98(20.29) | 82(19.48) |
| 3 | 295(61.08) | 259(61.52) |
| ACEI/ARB, No.(%) | 359(74.33) | 314(74.58) |
| Mono or dual antiplatelet, No.(%) | 478(98.96) | 416(98.81) |
| Statin, No.(%) | 472(97.72) | 414(98.34) |
| β-blockers, No.(%) | 419(86.75) | 367(87.17) |
| CCB, No.(%) | 117(24.22) | 103(24.47) |
| Anticoagulant, No.(%) | 12(2.48) | 9(2.14) |
| Furosemide, No.(%) | 57(11.80) | 45(10.69) |
| LVEF, % | 58.15±11.11 | 58.44±10.88 |
| PHQ-9 score^†^, point | 4.41±4.29 | 4.26±4.15 |
| Prevalence of depression, % | 187(38.72) | 160(38.00) |
| GAD-7 score^†^, point | 3.18±3.72 | 3.13±3.63 |
| Prevalence of anxiety, % | 125(25.88) | 109(25.89) |
| ^†^：scores were presented as means±SD，however were compared using Wilcoxonrank-sum test. | | |
| Abbreviation: EF: ejection fraction; CCR:creatinine clearance; ACEI: angiotensinconverting enzyme  inhibitor; HbA1c: glycosylated hemoglobin; hsCRP: high sensitivity C-reactive protein; hsTNT: high  sensitivity troponin T; ARB: angiotensin receptor blocker; CCB: Calcium channel blockers; e’: early  diastolic annular velocity; a’: late diastolic annular velocity; s’: systolic annular velocity; E/E’: transmitral to mitral annular early diastolic velocity ratio; | | |

# Supplementary Table 2

| **Table S2 Comparisons of baseline clinical characteristics between patients with LVEF ≥50% and <50%.** | | | |
| --- | --- | --- | --- |
|  | EF≥50% N=387 | EF<50% N=96 | p value |
| Age, y | 63.49±9.91 | 64.11±10.50 | 0.59 |
| Male, No.(%) | 292(75.45) | 78(81.25) | 0.23 |
| CCR, ml/min | 66.88±21.31 | 57.03±20.53 | **<.001** |
| LDLC, mmol/L | 2.87±0.91 | 2.85±0.85 | 0.89 |
| HDLC, mmol/L | 0.97±0.21 | 0.93±0.22 | 0.10 |
| HbA1c, % | 6.56±1.34 | 7.35±2.13 | **<.001** |
| Hypertension, No.(%) | 245(63.31) | 58(60.42) | 0.60 |
| Diabetes, No.(%) | 127(32.82) | 44(45.83) | **.017** |
| HsCRP, μg/ml | 2.13(0.78,7.02) | 5.47(2.34,18.40) | **<.001** |
| HsTNT, pg/ml | 12(9,28) | 63(22,528) | **<.001** |
| Nt-pro-BNP, pg/ml | 126(51,347) | 1785(689,4682) | **<.001** |
| History of revascularization, No.(%) | 127(32.82) | 34(35.42) | 0.63 |
| Stenosis degree, No.(%) |  |  | .076 |
| 1 | 80(20.67) | 10(10.42) |  |
| 2 | 75(19.38) | 23(23.96) |  |
| 3 | 232(59.95) | 63(65.63) |  |
| ACEI/ARB, No.(%) | 280(72.35) | 79(82.29) | **.046** |
| Mono or dual antiplatelet, No.(%) | 382(98.71) | 96(100) | 0.59 |
| Statin, No.(%) | 378(97.67) | 94(97.92) | >0.99 |
| β-blockers, No.(%) | 333(86.05) | 86(89.58) | 0.36 |
| CCB, No.(%) | 102(26.36) | 15(15.63) | **.028** |
| Anticoagulant, No.(%) | 10(2.58) | 2(2.08) | >0.99 |
| Furosemide, No.(%) | 17(4.39) | 40(41.67) | **<.001** |
| LVEF, % | 62.84±5.34 | 39.20±7.45 | **<.001** |
| PHQ-9 score†, point | 4.37±4.30 | 4.57±4.28 | 0.50 |
| Prevalence of depression, % | 142(36.69) | 45(46.88) | .067 |
| GAD-7 score†, point | 3.26±3.71 | 2.84±3.73 | 0.18 |
| Prevalence of anxiety, % | 106(27.39) | 19(19.79) | 0.13 |
| †：scores were presented as means±SD，however were compared using Wilcoxonrank-sum test. | | | |
| Abbreviation: EF: ejection fraction; CCR:creatinine clearance; ACEI: angiotensinconverting enzyme inhibitor; HbA1c: glycosylated hemoglobin; hsCRP: high sensitivity C-reactive protein; hsTNT: high sensitivity troponin T; ARB: angiotensin receptor blocker; CCB: Calcium channel blockers; e’: early diastolic annular velocity; a’: late diastolic annular velocity; s’: systolic annular velocity; E/E’: transmitral to mitral annular early diastolic velocity ratio; | | | |

# Supplementary Table 3

| **Table S3. Predictors for noncardiac rehospitalization in patients with different systolic function using Cox regression models^†^.** | | | | |
| --- | --- | --- | --- | --- |
| Variable | Crude | | Multivariable | |
|  | HR (95% CI) | P value | HR (95% CI) | P value |
| LVEF≥50% |  |  |  |  |
| Depression |  |  |  |  |
| PHQ-9 <5 | Ref. |  | Ref. |  |
| PHQ-9≥5 | 1.99 (1.05,3.76) | .034 | 1.78 (0.93,3.41) | .085 |
| CCR (every 1 ml/min increase) | 0.98(0.97,1.00) | .035 | 0.99 (0.97,1.00) | .081 |
| All patients |  |  |  |  |
| Depression |  |  |  |  |
| PHQ-9 <5 | Ref. |  | Ref. |  |
| PHQ-9≥5 | 1.65 (0.98, 2.78) | .062 | 1.44 (0.78,2.64) | 0.25 |
| Sex (male vs. female) | 0.57 (0.33, 0.98) | .043 | 0.64 (0.33,1.21) | 0.17 |
| CCR (every 1 ml/min increase) | 0.98 (0.96, 0.99) | <0.001 | 0.98 (0.97,1.00) | .057 |
| LVEF (per 1% increase) | 0.97 (0.95, 0.99) | 0.005 | 0.98 (0.95,1.00) | .045 |
| ^†^: Cox regression models with forward selection method (sle=0.25, sls=0.25) were used. | | | | |
| Abbreviation: MACE: major adverse cardiovascular event; HR: hazard ratio; CI: confidential interval. | | | | |

# Supplementary Table 4

| **Table S4. Predictors for composite outcome in patients with different systolic function using Cox regression models^†^.** | | | | |
| --- | --- | --- | --- | --- |
| Variable | Crude | | Multivariable | |
|  | HR (95% CI) | P value | HR (95% CI) | P value |
| LVEF≥50% |  |  |  |  |
| Depression |  |  |  |  |
| PHQ-9 <5 | Ref. |  | Ref. |  |
| PHQ-9≥5 | 1.75 (1.18, 2.60) | .005 | 1.68 (1.12,2.51) | .012 |
| Sex (male vs. female) | 0.69 (0.45, 1.06) | .088 | 0.63 (0.40,0.99) | .047 |
| HDLC (per 0.1mmol/L increase) | 0.28 (0.10 ,0.76) | .013 | 0.21 (0.08,0.57) | .002 |
| All patients |  |  |  |  |
| Depression |  |  |  |  |
| PHQ-9 <5 | Ref. |  | Ref. |  |
| PHQ-9≥5 | 1.46 (1.04,2.03) | .027 | 1.34 (0.93,1.95) | 0.12 |
| Sex (male vs. female) | 0.69 (0.48, 1.00) | .049 | 0.59 (0.39,0.90) | .014 |
| HDLC (per 1.0 mmol/L increase) | 0.42 (0.19 ,0.95) | .038 | 0.37 (0.15,0.90) | .029 |
| LVEF (per 1% increase) | 0.98 (0.96,0.99) | <0.001 | 0.97 (0.96,0.99) | <0.001 |
| ^†^: Cox regression models with forward selection method (sle=0.15, sls=0.15) were used. | | | | |
| Abbreviation: MACE: major adverse cardiovascular event; HR: hazard ratio; CI: confidential interval. | | | | |

| Supplementary Table 5 **Table S5. Comparisons of predictive values for noncardiac rehospitalization in patients with LVEF≥50% and in the whole population.** | | | | |
| --- | --- | --- | --- | --- |
|  | ROC Association Statistics | | | |
|  | AUC | standard error | 95% CI | *p* value |
| LVEF≥50% |  |  |  |  |
| Depression | 0.60 | .045 | 0.51,0.69 | ref |
| Creatinine clearance | 0.59 | .049 | 0.49,0.68 | 0.81 |
| Depression + Creatinine clearance | 0.64 | .050 | 0.54,0.73 | 0.17 |
| All patients |  |  |  |  |
| Depression | 0.61 | .043 | 0.53,0.70 | ref |
| Sex (male vs. female) | 0.56 | .042 | 0.48,0.64 | 0.33 |
| Creatinine clearance | 0.62 | .048 | 0.52,0.71 | 0.93 |
| LVEF | 0.49 | .051 | 0.48,0.64 | .069 |
| Depression + Sex + Creatinine clearance + LVEF | 0.66 | .048 | 0.56,0.75 | 0.11 |
| Abbreviation: MACE: major adverse cardiovascular event; LVEF: left ventricular ejection fraction; HDLC: high-density lipoprotein; ROC: Receiver Operating Characteristic Curve; AUC: area under curve; CI: confidential interval. | | | | |

| Supplementary Table 6 **Table S6. Comparisons of predictive values for composite in patients with LVEF≥50% and in the whole population.** | | | | |
| --- | --- | --- | --- | --- |
|  | ROC Association Statistics | | | |
|  | AUC | standard error | 95% CI | *p* value |
| LVEF≥50% |  |  |  |  |
| Depression | 0.60 | .032 | 0.54,0.66 | ref |
| HDLC | 0.59 | .036 | 0.52,0.66 | 0.83 |
| Sex | 0.54 | .029 | 0.48,0.60 | 0.11 |
| Depression + HDLC + Sex | 0.65 | .037 | 0.57,0.72 | .053 |
| All patients |  |  |  |  |
| Depression | 0.58 | .028 | 0.52,0.63 | ref |
| HDLC | 0.58 | .032 | 0.52,0.64 | 0.91 |
| Sex | 0.53 | .025 | 0.48,0.58 | 0.15 |
| LVEF | 0.61 | .033 | 0.55,0.67 | 0.44 |
| Depression + HDLC + Sex + LVEF | 0.67 | .032 | 0.61,0.73 | .003 |
| Abbreviation: MACE: major adverse cardiovascular event; LVEF: left ventricular ejection fraction; HDLC: high-density lipoprotein; ROC: Receiver Operating Characteristic Curve; AUC: area under curve; CI: confidential interval. | | | | |

# Supplementary Figure 1


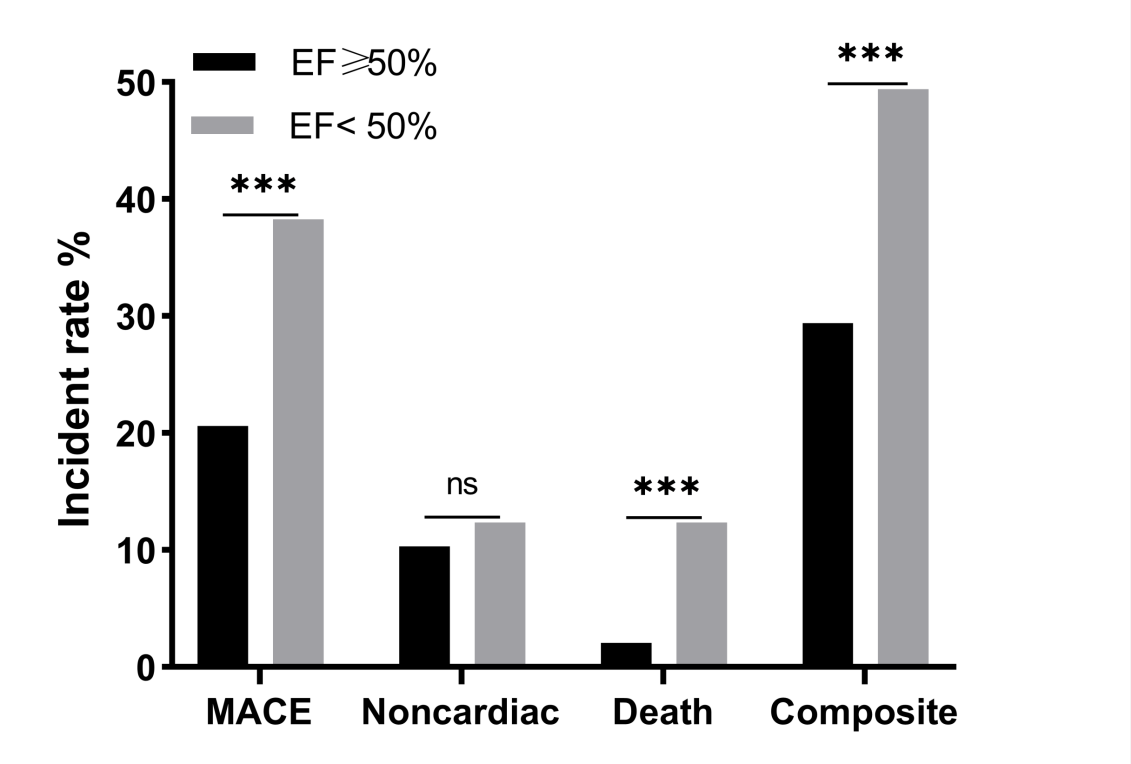


**Figure S1.** Comparison of clinical outcomes in obstructive CAD patients with LVEF≥50% and <50%.
